# Enhanced insecticidal efficacy of nanoencapsulated bergamot essential oil against stored-product pest *Tribolium castaneum* (Herbst)

**DOI:** 10.1038/s41598-026-61299-8

**Published:** 2026-07-17

**Authors:** Samar Sayed Ibrahim

**Affiliations:** Pests and Plant Protection Department, National Research Centre, 33 El-Buhouth Street, Dokki, Giza 12622 Egypt

**Keywords:** Bergamot essential oil, Feeding deterrence, Freeze-drying, Nanoencapsulation, Progeny inhibition, Stored-product pests, Biochemistry, Biological techniques, Biotechnology, Chemistry, Environmental sciences, Plant sciences

## Abstract

In this study, bergamot essential oil (BEO) was encapsulated within β-cyclodextrin/gum Arabic (BEO–βCD/GA) to enhance its physicochemical properties and its toxicity against *Tribolium castaneum*, a major stored-product pest. Gas chromatography–mass spectrometry detected D-limonene (59.38%) as the predominant component of BEO. The optimized BEO–βCD/GA exhibited a particle size of 194.26 ± 2.45 nm, a low polydispersity index (0.04 ± 0.003), zeta potential of − 31.23 mV, and a high encapsulation efficiency of 88.12%, indicating a homogeneous system. BEO–βCD/GA showed greater toxicity than BEO, producing lower LC values and higher *T. castaneum* mortality across concentrations and exposure periods. The carrier substances showed no toxicity to *T. castaneum*, confirming that the observed effects were due to BEO or BEO–βCD/GA. The LC_50_ and LC_90_ values of BEO–βCD/GA were consistently lower than those of BEO across all exposure periods (1–7 days). At 7 days, BEO–βCD/GA recorded markedly lower LC_50_ (106.85 ppm; 3.15–173.54) and LC_90_ (319.49 ppm; 200.18–4468.97) compared with BEO (LC_50_: 437.72 ppm: 359.29–596.06; LC_90_: 3195.87 ppm: 1710.83–10801.66). The BEO–βCD/GA achieved near-complete inhibition of progeny emergence (97–100%) at higher concentrations, compared to 61–79% for BEO. In addition, BEO–βCD/GA significantly reduced grain weight loss and exhibited strong antifeedant activity. Neither treatment adversely affected wheat germination nor seedling vigor. These physicochemical characteristics, together with the formulation’s enhanced insecticidal performance, suggest improved stability and bioavailability of BEO within the βCD/GA system, making it a promising, eco-friendly alternative for sustainable stored-product pest management.

**Supplementary Information:**

The online version contains supplementary material available at 10.1038/s41598-026-61299-8.

## Introduction

The red flour beetle, *Tribolium castaneum* (Herbst) (Coleoptera: Tenebrionidae), is a highly destructive pest of stored food products, notably affecting sorghum, rice, maize, and wheat-derived commodities^1^. Its infestation results in significant losses in both quality and quantity, including contamination, deterioration of grain quality, and reduction in germination capacity. The insect demonstrates remarkable adaptability, showing tolerance to low humidity, a longer lifespan, and the ability to endure adverse environments^2^.

To control *T. castaneum*, conventional pest management techniques, including chemical insecticides and fumigants, have been widely employed. However, the widespread and irresponsible use of these substances has led to the emergence of resistance, decreased effectiveness, and major health and environmental issues^3^. Residual toxicity in food products and hazards to non-target organisms further constrain their sustainability.

Essential oils derived from plants have emerged as effective bioinsecticides owing to their biodegradability, low mammalian toxicity, and multiple mechanisms of action, including contact toxicity, fumigant properties, repellency, and feeding deterrents^4^. Several studies have evidenced the efficacy of essential oils against *T. castaneum*^1–3^, underscoring their potential as natural pest-control agents. Despite these advantageous properties, the practical application of essential oils is constrained by their significant volatility, poor water solubility, and limited stability, which diminishes their effectiveness as biopesticides^5^. To address these constraints, nanotechnology-based delivery technologies have been progressively investigated.

Formulating essential oils into nanosystems, such as nanoemulsions and nanocapsules, enhances their efficiency, stability, controlled release, and bioavailability^6^. Nanoformulations have been reported to enhance the insecticidal effects of essential oils against various stored-product insects, including *T. castaneum*^5,7–9^, demonstrating a greater potency than the non-formulated oils.

Among plant-derived compounds, bergamot (*Citrus bergamia* Risso et Poiteau; F: Rutaceae) is an essential oil widely used in the perfumery and fragrance industries for its appealing aroma. It possesses significant uses in the pharmaceutical and food industries. Recognized as safe, it is frequently utilized as a flavoring agent in a wide range of food and beverage products^10^. Bergamot essential oil predominantly consists of volatile components (about 93–96%), primarily terpenes such as limonene, linalool, and linalyl acetate, which contribute to its distinctive fragrance and functional properties. It also comprises a minor proportion (4–7%) of non-volatile chemicals, including bergamottin, citropten, and bergapten, which enhance its biological activity^11^. Insecticidal properties of BEO, attributed to its bioactive constituents, have been reported^12–14^.

The utilization of natural carrier systems such as β-cyclodextrin and gum Arabic provides an efficient method to enhance encapsulation efficiency, stability, and controlled release of essential oils, hence augmenting their biological activity. β-cyclodextrins are non-toxic and relatively inexpensive compounds; owing to their unique molecular structure, they can form inclusion complexes with various compounds^15^. Gum Arabic is a polysaccharide with minor protein content, which enhances its role as a natural emulsifier and stabilizing agent, successfully preserving stability at the oil-water interface^16^. Prior studies have documented the encapsulation of essential oils, such as bergamot, saffron, *Eucalyptus staigeriana*, and cinnamon, utilizing carriers such as maltodextrin, β-cyclodextrin, and gum Arabic, either individually or in combination^15,17–19^.

In this study, bergamot oil was encapsulated using a combination of β-cyclodextrin and gum Arabic through freeze-drying, an approach that enhances its thermal stability and prolongs its efficacy^20^. Unlike conventional applications, this strategy enables more sustained pest control, potentially reducing required dosage and environmental impact. While β-cyclodextrin and gum Arabic have been utilized as encapsulating agents for essential oils, the use of bergamot essential oil encapsulated in a β-CD/GA system for *T. castaneum* control, alongside the concurrent evaluation of insect mortality, progeny suppression, feeding deterrence, grain weight loss, seed viability, and germination, has not been documented previously. Therefore, the present study aimed to (i) synthesize and characterize BEO-βCD/GA inclusion complexes, (ii) evaluate the insecticidal activity of pure and nanoformulated BEO against *T. castaneum*, and (iii) assess their effectiveness in protecting stored grains and preserving seed quality.

## Materials and methods

### Chemicals and materials

Bergamot, *Citrus bergamia* (Risso et Poiteau), essential oil was obtained commercially from the essential oil extraction unit of the National Research Centre (NRC), Egypt. Beta-Cyclodextrin (β-CD: Purity: 97%, Molecular Weight: 1134.98) and gum acacia powder AR (gum Arabic) were acquired from Alpha Chemika (India). HPLC-grade solvents were purchased from Sigma-Aldrich. Talcum powder was purchased from the local market. Soft wheat flour and wheat (*Triticum aestivum* L.) grains used for biological bioassays and insect feeding were purchased from a nearby market and stored at -4 °C for at least 5 days before use in order to eliminate any living insects in the products.

### Phytochemical screening of bergamot essential oil

Phytochemical analysis of bergamot essential oil was performed using gas chromatography-mass spectrometry. The oil sample was dissolved in dichloromethane and injected into the GC. The GC-MS system (Agilent Technologies) was equipped with a gas chromatograph (7890B) and mass spectrometer detector (5977 A) at the Central Laboratories Network, NRC, Egypt. The GC was equipped with an HP-5MS column (15 m × 0.25 mm internal diameter, 0.25 μm film thickness). Analyses were carried out using hydrogen as the carrier gas at a flow rate of 1.1 mL/min at a splitless injection volume of 1.0 µL and the following temperature program: 40 °C for 1 min; rising at 10 °C/min to 200 °C and held for 1 min; rising at 20 °C/min to 220 °C and held for 1 min; rising at 30 °C/min to 300 °C and held for 3 min. The system was operated under the manufacturer’s recommended conditions for hydrogen carrier gas. The injector and detector were held at 250 °C and 300 °C, respectively. Mass spectra were obtained by electron ionization (EI) at 70 eV, over the m/z range 33–600, with a solvent delay of 1.60 min. The mass temperature was 230 °C, and the Quad was 150 °C. Identification of the different constituents was determined by comparing the fragmentation patterns of the spectra with those stored in the Wiley and NIST Mass Spectral Libraries.

### Synthesis of bergamot essential oil–loaded β-cyclodextrin/gum Arabic nanocomplexes (BEO–βCD/GA)

Bergamot essential oil (BEO) was encapsulated in β-cyclodextrin (βCD) and gum Arabic (GA) using an emulsification and freeze-drying method^21^. BEO was used as the core material at concentrations of 10.0%, 5.0%, and 2.5%, while a βCD-GA mixture (1:1, w/w) served as the wall material. BEO: water: βCD/GA ratios of 10:50:40, 5:50:45, and 2.5:50:47.5 (w/w) were used to prepare three formulations (F1, F2, and F3). First, βCD and GA were dissolved in distilled water and heated for 45 min at 60 °C. After that, the solutions were covered and left overnight at room temperature. Each BEO concentration was added separately to the respective βCD/GA solution after 24 h.

To produce stable emulsions, the resultant mixtures were placed in containers immersed in an ice bath and homogenized using ultrasonication (Ningbo Haishu Kesheng Ultrasonic Equipment Co., Ltd., China) at 70% amplitude for five minutes. After 20 h of freezing at − 20 °C, the emulsions were lyophilized for 24 h at − 50 °C in a Christ Lab freeze dryer. Following freeze-drying, the resulting solids were crushed to a fine powder, and the BEO–βCD/GA formulations were stored at 4 °C until bioassays and further characterization. A control formulation was prepared following the same procedure but without the addition of bergamot essential oil.

### Physicochemical characterization of BEO–βCD/GA nanocapsules

#### Dynamic light scattering (DLS) measurements

The particle size, polydispersity index (PDI), and zeta potential of BEO-βCD/GA inclusions were measured by dynamic light scattering (DLS) using Malvern Zetasizer Nano-ZS (Malvern, Worcestershire, UK). Ten milliliters of distilled water were used to suspend approximately 0.2 g of each concentration under continuous magnetic stirring for thirty minutes^14^. After two minutes of equilibration, samples were measured at 25 °C, and each measurement was conducted in triplicate.

#### Encapsulation efficiency (%EE)

The amount of BEO encapsulated into the βCD/GA complex was determined to calculate the encapsulation efficiency (EE). Encapsulation efficiency was estimated using an indirect extraction method based on the complete dissolution of the inclusion complexes^22,23^. Briefly, 0.1 g of BEO–βCD/GA inclusion complexes synthesized at 10.0, 5.0, and 2.5% was dissolved in 2 mL of absolute ethanol. Each sample was placed in a sealed centrifuge tube and heated to 50 °C for 30 min to guarantee total dissolution. A UV–Vis spectrophotometer (T80 + UV/VIS Spectrophotometer, PG Instruments Ltd.) was employed to measure the absorbance of the resultant solutions at 310 nm, corresponding to one of the characteristic maximum absorbance wavelengths of BEO, as previously reported^15^. A calibration curve prepared from a series of standard BEO solutions in absolute ethanol, measured at 310 nm, was used to determine the concentration of bergamot essential oil. Every measurement was carried out three times. The following formula was used to determine the encapsulation efficiency^24^:

$$\:\mathrm{\%}\mathrm{E}\mathrm{E}=\frac{A-B}{A} \times \:100$$

A: the total amount of bergamot essential oil initially added, and B: the amount of the oil measured in the supernatant.

The analytical curve that was used for measurements is as follows:

$$\:y=0.01\:x+0.0384\:(\mathrm{R}^{2}=0.998)$$

x = the concentration, y = the absorbance at 310 nm.

### Insect

*Tribolium castaneum* adults were maintained for multiple generations for over 3 years in the laboratory of the Pests and Plant Protection Department at NRC. Insects were reared on soft wheat flour plus 5% of the brewer’s yeast (by weight) in glass jars (2 L). The jars were covered with fine muslin cloth to allow for ventilation. About 300 unsexed adults were placed in glass jars with 500 g of rearing medium in order to obtain *T. castaneum* adults of the same age. All adults were removed following a 5-day oviposition period. After allowing the progeny to develop, newly emerged adults aged < 7 days were used in the subsequent experiments. The insects and experiments were maintained in a laboratory under continuous darkness at 30 ± 2 °C and 65 ± 5% relative humidity^25^.

### Bioassays

#### Insecticidal toxicity

This investigation aimed to assess the toxicity of non-formulated BEO and the optimized BEO–βCD/GA inclusion with the highest EE% (F1) against *T. castaneum* adults under dietary exposure conditions. Airtight glass containers (200 mL) were provided with 20 g of broken wheat grains, mixed with BEO–βCD/GA powder at varying concentrations (500, 400, 300, 200, and 100 ppm) at respective rates of 10, 8, 6, 4, and 2 mg/20 g. To evaluate the toxicity of pure BEO, 10.0 g of the oil was blended with 90 g of a commercial carrier (talcum powder) (w/w) and then allowed to dry at room temperature^26^. The same concentration levels of 500, 400, 300, 200, and 100 ppm were utilized. To evaluate the potential adverse effects of the carrier substances used, a control βCD/GA inclusion and talcum powder alone (free of oil) were used at the same concentrations. The control group did not administer any powder treatment. Wheat grains subjected to different treatments were agitated vigorously to ensure uniform distribution of the powder. Ten individual adult *T. castaneum* insects were released separately. All treatments were conducted in quintuplicate, and mortality was recorded after 1, 3, 5, and 7 days. Four groups were conducted separately for each time interval. Insects that exhibited immobility following disturbance were deemed dead. The number of dead insects was recorded to determine the lethal concentration values. Because there was no significant difference between the positive control (βCD/GA inclusion or talcum powder) and the negative control (no powder treatment), only the negative control was used in subsequent experiments.

#### Progeny production

Following the completion of the final mortality assessment of *T. castaneum* after 7 days of exposure, all individuals, including dead and surviving adults, were taken out of the containers, and then the treatments were maintained under the previously specified controlled conditions. Progeny production was determined by counting the emerging adults in each replicate at 30, 35, 40, and 45 days post-removal of the parental insects. At each observation interval, newly emerged adults were counted and subsequently removed to avoid double-counting. The inhibition rate percentage (IR%) was calculated as follows:

$$\:\mathrm{I}\mathrm{R}\mathrm{\%}=\frac{\left(\mathrm{M}\mathrm{e}\mathrm{a}\mathrm{n}\:\mathrm{N}\mathrm{o}.\:\mathrm{o}\mathrm{f}\:\mathrm{c}\mathrm{o}\mathrm{n}\mathrm{t}\mathrm{r}\mathrm{o}\mathrm{l}\:\mathrm{i}\mathrm{n}\mathrm{s}\mathrm{e}\mathrm{c}\mathrm{t}\mathrm{s}-\mathrm{M}\mathrm{e}\mathrm{a}\mathrm{n}\:\mathrm{N}\mathrm{o}.\:\mathrm{o}\mathrm{f}\:\mathrm{t}\mathrm{r}\mathrm{e}\mathrm{a}\mathrm{t}\mathrm{e}\mathrm{d}\:\mathrm{i}\mathrm{n}\mathrm{s}\mathrm{e}\mathrm{c}\mathrm{t}\mathrm{s}\right)}{\mathrm{M}\mathrm{e}\mathrm{a}\mathrm{n}\:\mathrm{N}\mathrm{o}.\:\mathrm{o}\mathrm{f}\:\mathrm{c}\mathrm{o}\mathrm{n}\mathrm{t}\mathrm{r}\mathrm{o}\mathrm{l}\:\mathrm{i}\mathrm{n}\mathrm{s}\mathrm{e}\mathrm{c}\mathrm{t}\mathrm{s}} \times 100$$

#### Grain weight loss (%) and antifeedant activity

The antifeedant efficacy of BEO and the optimized BEO–βCD/GA inclusion (F1) against *T. castaneum* adults, along with the assessment of the weight-loss percentage induced by the treatments, were examined. Wheat grains were subjected to sub-lethal and lethal concentrations (LC_25_, LC_50_, and LC_90_ for a 7-day exposure interval) using the previously described method. One kg of wheat grain was treated with selected concentrations of BEO and BEO–βCD/GA inclusion and stored in sealed airtight plastic bags (2 L) (25 × 27.2 cm). Five replicates were conducted for each treatment, and 50 adult insects were released in each replicate. Another group supplied with untreated wheat grains served as a control. All bags were then maintained for 3 months. The percentage of weight loss in the grains was determined using the subsequent equation:

$$\:\mathrm{W}\mathrm{e}\mathrm{i}\mathrm{g}\mathrm{h}\mathrm{t}\:\mathrm{l}\mathrm{o}\mathrm{s}\mathrm{s}\:\mathrm{p}\mathrm{e}\mathrm{c}\mathrm{e}\mathrm{n}\mathrm{t}\mathrm{a}\mathrm{g}\mathrm{e}=\frac{\left(W1-W2\right)}{W1} \times 100$$

Where W1 represents the weight of wheat grains prior to the experiment, and W2 represents the weight of the wheat grains following the storage duration.

Accordingly, the antifeeding action of BEO and BEO–βCD/GA inclusion was assessed by calculating the feeding deterrent index (FDI %) using the following equation^27^:

$$\:\mathrm{F}\mathrm{D}\mathrm{I}\mathrm{\%}=\frac{\left(C-T\right)}{C} \times 100$$

Where C represents the weight loss in the control group, and T indicates the weight loss in the treatment group.

#### The effect on the germination of wheat seeds

Wheat grains subjected to lethal concentration (LC_90_ of a 7-day exposure period) of BEO and the optimized BEO–βCD/GA inclusion (F1) have been examined for viability and germination percentage. Wheat grains were treated as previously described and stored for 7 days; then, 10 grains were distributed on two layers of filter paper medium soaked with distilled water in an 11 cm Petri dish for each replicate. Each treatment was performed in five replicates, and five additional replicates were maintained for untreated grains as a control group. The experiment was designed as an initial phytotoxicity screening and therefore utilized a limited number of seeds per replicate, providing a preliminary assessment of treatment effects on seed viability.

To allow the wheat grains to germinate, they were kept in the dark at 22 °C for the first 2–3 days. After the radicle emerged, the seedlings were moved to a controlled photoperiod (12 h of light and 12 h of dark) for the remainder of the 7-day experiment. Germination percentage was recorded, and root and shoot lengths were measured at the end of the experiment in accordance with the International Seed Testing Association (ISTA) regulations. The following equations^28^ were used:

$$\:\mathrm{G}\mathrm{i}=\frac{GT\mathrm{\%}}{GC\%}$$

Gi: germination index; %Gt: % germination in treatment; %Gc: % germination in control.

$$\:\mathrm{V}\mathrm{i}=(Arl+Asl) \times \%G$$

Vi: Seedling vigor index; Arl: Average root length; Asl: Average shoot length; %G: % seed germination.

### Statistical analysis

All data were subjected to analysis of variance (ANOVA). Differences among treatment means were compared using Tukey’s honestly significant difference (HSD) test at the *p* < 0.05 significance level. Lethal concentration values and their corresponding confidence intervals were estimated using probit analysis according to Finney’s method^29^. The feeding deterrent index (FDI%) of bergamot essential oil (BEO) and its nanoformulation (BEO–βCD/GA) was analyzed using an independent samples t-test. All bioassay experiments were independently replicated twice to verify reproducibility. Due to the absence of significant differences between the two runs, the data were combined and analyzed statistically. All data were analyzed using IBM SPSS Statistics (version 20; IBM Corp., Armonk, NY, USA).

## Results

### GC-MS analysis

**Fig. 1:**
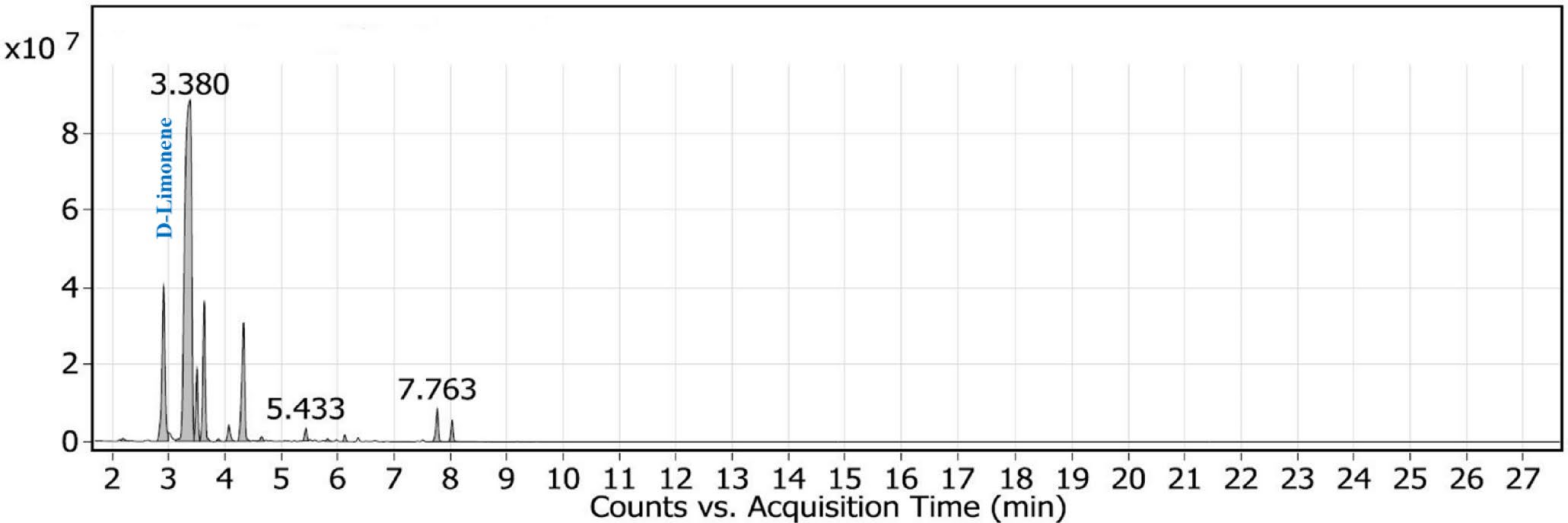
GC-MS chromatogram of bergamot essential oil phytochemicals.

The phytochemical screening of bergamot essential oil by GC-MS identified 14 compounds (Table 1; Fig. 1). D-limonene (59.38%) was the main compound, followed by β-myrcene (12.53%), linalool (9.4%), and trans-β-ocimene (8.61%). Beta-ocimene, triacetin, and α-terpinolen, as well as other compounds, were identified at lower percentages.

**Table 1:** Main phytochemicals identified by gas chromatography-mass spectrometry (GC-MS).

Peak	RT^a^	Compound name	Formula	Area Sum^b^ %
1	2.189	α-Pinene	C10H16	0.23
2	2.91	β-Myrcene	C10H16	12.53
3	3.38	D-Limonene	C10H16	59.38
4	3.502	β-ocimene	C10H16	3.92
5	3.631	trans-β-ocimene	C10H16	8.61
6	3.882	2-Furanmethanol, 5-ethenyltetrahydro-.alpha.,.alpha.,5-trimethyl-, cis-	C10H18O2	0.12
7	4.069	Cyclohexene, 1-methyl-4-(1-methylethylidene)-	C10H16	1.09
8	4.333	Linalool	C10H18O	9.4
9	4.648	2,4,6-Octatriene, 2,6-dimethyl-, (E, E)-	C10H16	0.29
10	5.433	α-Terpineol	C10H18O	0.74
11	5.819	trans-Verbenol	C10H16O	0.15
12	6.122	(-)-Carvone	C10H14O	0.33
13	7.763	Triacetin	C9H14O6	2
14	8.027	Linalyl acetate	C12H20O2	1.21

RT^a^: Retention time (min), Area Sum^b^: Peak area/Total peak area.

### Characterization of BEO–βCD/GA inclusion

**Fig. 2:**
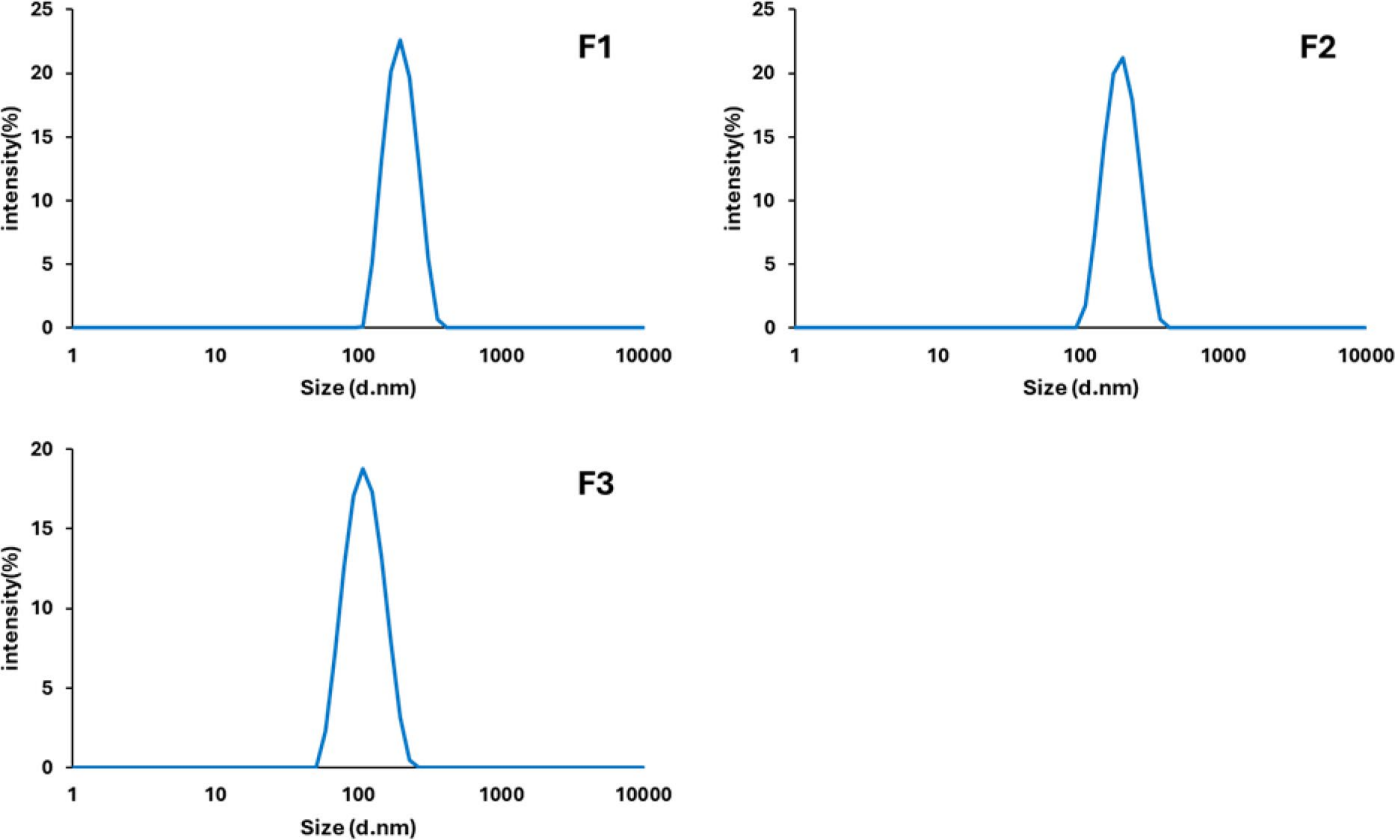
Size distribution by intensity of different formulations (F1, F2, and F3) of bergamot essential oil–loaded β-Cyclodextrin/Gum Arabic (BEO–βCD/GA). F1, F2, and F3 are formulations that contain 10.0, 5.0, and 2.5% bergamot oil.

The average size distribution and polydispersity index (PDI) of the prepared formulations (F1, F2, and F3) varied with BEO concentration (Table 2; Fig. 2). There were statistical differences in the particle size among the prepared formulations. F3 showed the smallest particle size (105.46 ± 0.55 nm) and differed significantly (*p* < 0.05) from F1 (194.26 ± 2.45 nm) and F2 (187.13 ± 2.31 nm), whereas no significant difference was observed between F1 and F2 (*p* > 0.05). The homogeneity and monodispersity of the produced BEO–βCD/GA inclusions are confirmed by the very low polydispersity index (PDI < 0.06) obtained for all formulations, which implies a highly uniform particle size distribution. The zeta potential measurements revealed significant variation among formulations. The highest negative surface charge was observed for F1 (− 31.23 mV), whereas F2 and F3 exhibited zeta potential values of − 19.06 and − 22.40 mV, respectively (Table 2, Fig. S1).

**Table 2:** Particle size, polydispersity index (PDI), zeta potential, and encapsulation efficiency percentage (EE%) of different formulations (F1, F2, and F3) of bergamot essential oil–loaded β-Cyclodextrin/Gum Arabic nanocomplexes.

Formulation	Particle size (d.nm)	PDI	Zeta potential (mV)	EE%
F1*	194.26 ± 2.45a	0.0450 ± 0.003a	-31.23 ± 0.49c	88.12 ± 0.15a
F2	187.13 ± 2.31a	0.0240 ± 0.018a	-19.06 ± 0.08a	70.64 ± 0.57b
F3	105.46 ± 0.55b	0.0640 ± 0.010a	-22.4 ± 0.10b	53.82 ± 0.22c
F_(df1,df2)_, *P*	623.64_(2,6)_, *p* < 0.001	2.66_(2,6)_, *p* = 0.14	458.04_(2,6)_, *p* < 0.001	2171.68_(2,6)_, *p* < 0.001

* F1, F2, and F3 are formulations that contain 10.0, 5.0, and 2.5% bergamot oil. Means (± SE) followed by the same letters within the same column are not significantly different (Tukey’s HSD test, *p* < 0.05).

One important parameter for evaluating the effectiveness of the produced nanocapsule delivery systems is the encapsulation efficiency. The EE% of BEO–βCD/GA inclusions was significantly different with different concentrations of BEO (Table 2). EE% increased with increasing oil concentration, reaching 88.12% for F1 containing 10.0% BEO, followed by F2 (70.64%) and F3 (53.82%) containing 5.0% and 2.5% BEO, respectively.

### Toxicity study

**Fig. 3:**
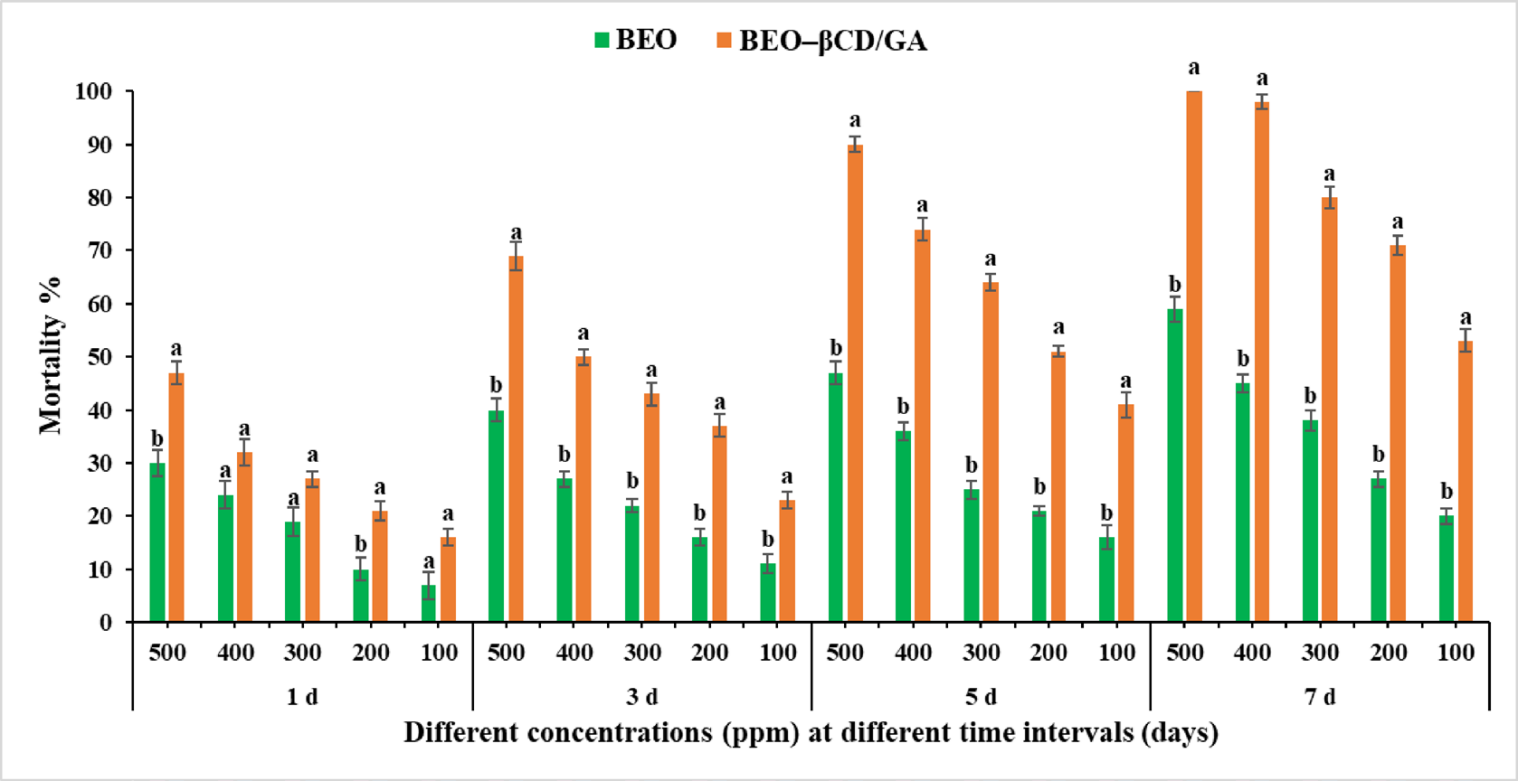
Percentage mortality of *Tribolium castaneum* adults after treatment with bergamot essential oil (BEO) and bergamot essential oil–loaded β-Cyclodextrin/Gum Arabic (BEO–βCD/GA) at 4 exposure time intervals: 1, 3, 5, and 7 days. Values are expressed as mean ± standard error (M ± SE). Different lowercase letters indicate significant differences between pure and nano-formulated oil at the same concentration and exposure time (*p* < 0.05; ANOVA followed by Tukey’s HSD test).

In the toxicity test, the mortality rate of adult *T. castaneum* increased with increasing concentration and duration of exposure to BEO and BEO–βCD/GA (Fig. 3). Across all exposure durations (1, 3, 5, and 7 days), the BEO–βCD/GA showed significantly higher mortality rates than the pure oil at the same concentrations. After one day, mortality varied from 7% to 30% for BEO and from 16% to 47% for BEO–βCD/GA. A notable increase in mortality was observed for BEO–βCD/GA at 500 and 200 ppm compared with the pure oil, although no significant differences were observed at other concentrations (F_(df)_: 36.39_(10,99)_; *p* < 0.001). At 3, 5, and 7 days, BEO–βCD/GA showed significantly higher mortality across all tested concentrations, indicating increased insecticidal efficacy. After three days of exposure, mortality significantly increased, attaining 11–40% for BEO and 23–69% for BEO–βCD/GA (F_(df)_: 117.20_(10,99)_; *p* < 0.001). After five days, mortality increased to 16–47% for BEO and 41–90% with BEO–βCD/GA (F_(df)_: 233.89_(10,99)_; *p* < 0.001). At 7 days, the peak mortality rates were observed, varying from 20 to 59% for BEO and 53 to 100% for BEO–βCD/GA (F_(df)_: 336.83_(10,99)_; *p* < 0.001). Complete mortality (100%) was achieved with BEO–βCD/GA at 500 ppm. Significant differences were detected within the same treatment throughout different intervals.

The probit analysis demonstrated a reduction in LC values with prolonged exposure time for both treatments, signifying increased toxicity over time (Table 3). The BEO–βCD/GA showed greater toxicity than the pure oil, as evidenced by lower LC values. The LC_50_ values of the BEO–βCD/GA were approximately 1.5, 3.0, 4.6, and 4.1 times lower than those of BEO after 1, 3, 5, and 7 days of exposure, respectively. Correspondingly, LC_25_ levels decreased by approximately 1.9 to 3.2 times, whereas LC_90_ values showed the greatest variation, reaching up to 10 times in 7 days. The results demonstrate that BEO–βCD/GA markedly improves the insecticidal efficacy of bergamot essential oil, especially during extended exposure durations.

**Table 3:** Probit analysis of mortality in *Tribolium castaneum* adults exposed to bergamot essential oil (BEO) and bergamot essential oil–loaded β-Cyclodextrin/Gum Arabic (BEO–βCD/GA) at different exposure time intervals.

Exposure time (days)	Treatment	LC_25_ (95% Confidence Limit (LCL-UCL)	LC_50_ (95% Confidence Limit (LCL-UCL)	LC_90_ (95% Confidence Limit (LCL-UCL)	Slope (SE)	X^2^ (df)
1	BEO	418.12 (337.80-592.06)	1212.13 (777.92-3354.19)	9158.62 (3322.49-103401.91)	1.45 ± 0.30	1.04 (3)
	BEO–βCD/GA	223.32 (162.59-278.79)	788.53 (552.62-1716.16)	8665.97 (3131.31-97818.19)	1.23 ± 0.25	3.77 (3)
3	BEO	308.48 (247.07-394.13)	976.49 (659.01-2327.65)	8719.94 (3219.03-89756.03)	1.34 ± 0.27	2.61 (3)
	BEO–βCD/GA	121.30 (80.96–154.10)	327.47 (277.14-402.52)	2160.93 (1300.21-5510.09)	1.56 ± 0.24	4.76 (3)
5	BEO	221.13 (163.52-273.27)	736.16 (531.18-1450.44)	7234.42 (2843.46-60591.90)	1.29 ± 0.25	3.84 (3)
	BEO–βCD/GA	68.60 (1.86-128.79)	159.64 (38.49–241.50)	794.46 (439.06-22083.30)	1.83 ± 0.24	9.07 (3)
7	BEO	153.74 (107.49-190.73)	437.72 (359.29-596.06)	3195.87 (1710.83-10801.66)	1.48 ± 0.24	3.02 (3)
	BEO–βCD/GA	60.04 (0.09-116.16)	106.85 (3.15-173.54)	319.49 (200.18-4468.97)	2.69 ± 0.29	17.46 (3)

LC: lethal concentration (ppm); LCL: Lower confidence level; UCL: Upper confidence level; X^2^ (df): Chi-square value (degrees of freedom).

### Post-effect on progeny production

The post-effects of BEO and BEO–βCD/GA on progeny production of *T. castaneum* are presented in Table 4. The number of emerging adults dropped markedly with increasing concentration for both treatments. Nevertheless, the BEO–βCD/GA produced a significantly lower number of emerging adults than the pure oil at all evaluated concentrations. At the maximum level of 500 ppm, BEO yielded 43.70 ± 1.74 adults, while BEO–βCD/GA exhibited no adult emergence (0.00 ± 0.00). At the lowest concentration (100 ppm), the BEO–βCD/GA (61.80 ± 2.18 adults) considerably decreased progeny in comparison to BEO (161.20 ± 2.94 adults). The control treatment exhibited the greatest quantity of emerging adults (210.80 ± 2.73).

**Table 4:** Post-effects of bergamot essential oil (BEO) and bergamot essential oil–loaded β-Cyclodextrin/Gum Arabic (BEO–βCD/GA) on the average number of emerged adults and the inhibition rate percentage (IR%) of *Tribolium castaneum*.

Treatment	Concentration (ppm)	Emerged adults	%IR
BEO	500	43.70 ± 1.74f	79.13 ± 0.83c
	400	64.50 ± 1.92e	69.20 ± 0.92d
	300	82.00 ± 1.65d	60.84 ± 0.79e
	200	107.90 ± 2.34c	48.47 ± 1.12f
	100	161.20 ± 2.94b	23.02 ± 1.40 g
BEO–βCD/GA	500	0.00 ± 0.00 h	100.00 ± 0.00a
	400	3.90 ± 0.90 h	98.14 ± 0.43a
	300	7.10 ± 0.87 h	96.61 ± 0.42a
	200	26.90 ± 1.39 g	87.15 ± 0.66b
	100	61.80 ± 2.18e	70.49 ± 1.04d
Control	-	210.8 ± 2.73a	-
F_(df1,df2)_, *P*		1276.91_(10,99)_, *p* < 0.0001	830.55_(9,90)_, *p* < 0.0001

Means (± SE) followed by the same letters within the same column are not significantly different (Tukey’s HSD test, *p* < 0.05).

Accordingly, the inhibition rate (IR%) increased significantly with increasing concentration, and the BEO–βCD/GA consistently demonstrated greater inhibition than BEO. At 500 ppm, BEO–βCD/GA attained complete inhibition (100.00 ± 0.00%), while BEO exhibited 79.13 ± 0.83% inhibition. At the minimal concentration (100 ppm), BEO–βCD/GA attained 70.49 ± 1.04% inhibition, far surpassing the 23.02 ± 1.40% recorded for BEO.

### Grain weight loss and antifeedant activity

Table 5 presents the effects of BEO and BEO–βCD/GA on grain weight loss and feeding deterrence against *T. castaneum*. Grain weight loss decreased with rising concentration for both treatments. At LC_25_, the weight loss percentages were 3.81% for BEO and 3.38% for BEO–βCD/GA, compared with the control value of 4.42%. At elevated concentrations, significant reductions were observed. This tendency was more evident at LC_90_, where the BEO–βCD/GA exhibited nearly negligible weight loss (0.06%), in contrast to 1.26% for BEO.

**Table 5:** Grain weight loss (%wt loss) and feeding deterrence (% FDI) of bergamot essential oil (BEO) and bergamot essential oil–loaded β-Cyclodextrin/Gum Arabic (BEO–βCD/GA) against *Tribolium castaneum*.

	Concentration (ppm)					
	LC_25_		LC_50_		LC_90_	
Treatment	%wt loss	% FDI	%wt loss	% FDI	%wt loss	% FDI
BEO	3.81 ± 0.34ab	13.80 ± 7.70a	2.95 ± 0.25b	33.26 ± 5.59b	1.26 ± 0.19b	71.49 ± 4.22b
BEO–βCD/GA	3.38 ± 0.27b	23.53 ± 6.07a	0.97 ± 0.15c	78.05 ± 3.36a	0.06 ± 0.03c	98.64 ± 0.77a
Control	4.42 ± 0.22a	-	4.42 ± 0.22a	-	4.42 ± 0.22a	-
F_(df1,df2)_, *P*	3.43_(2,27)_, *p* = 0.04		67.31_(2,27)_, *P* < 0.0001		175.94_(2,27)_, *P* < 0.0001	
*t*_(df)_, *P*	-	-0.99_(18)_, *p* = 0.33	-	-6.87_(18)_, *p* < 0.0001	-	-6.33_(18)_, *p* < 0.0001

Means (± SE) followed by the same letters within the same column are not significantly different (Tukey’s HSD test, *p* < 0.05) (t test, *p* < 0.05).

Feeding deterrence increased significantly with concentration (*p* < 0.0001), and the BEO–βCD/GA consistently showed higher FDI values than the BEO. At LC_25_, FDI values were comparatively low and did not differ significantly between treatments. At LC_50_, the BEO–βCD/GA exhibited a much larger FDI of 78.05% compared with BEO’s 33.26%, with notable differences between the treatments. At LC_90_, BEO–βCD/GA attained nearly total feeding deterrence (98.64%), markedly surpassing BEO (71.49%). The results demonstrate a significant enhancement of antifeedant activity by the BEO–βCD/GA (Table 5).

### Seed germination

Table 6 illustrates the impact of BEO and BEO–βCD/GA at LC_90_ on wheat germination and seedling development. No notable variations were detected across treatments for germination percentage or seedling vigor index. Germination rates were consistently high across all treatments, varying from 89.00% (BEO) to 96.00% (control). The germination index exhibited minimal variation, recording values of 0.92 for BEO and 0.98 for BEO–βCD/GA. The seedling vigor index exhibited a comparable pattern, with values of 1731.60, 1900.30, and 1948.70 for BEO, BEO–βCD/GA, and the control group, respectively.

**Table 6:** Effects of bergamot essential oil (BEO) and bergamot essential oil–loaded β-Cyclodextrin/Gum Arabic (BEO–βCD/GA) at LC_90_ on wheat germination and development.

Treatment	Germination %	Germination index	Seedling vigor index
BEO	89.00 ± 3.14a	0.92	1731.60 ± 75.61a
BEO–βCD/GA	95.00 ± 1.67a	0.98	1900.30 ± 45.76a
Control	96.00 ± 2.21a	1	1948.70 ± 78.59a
F_(df1,df2)_, *P*	2.44_(2,27)_, *p* = 0.10	-	2.78_(2,27)_, *p* = 0.07

Means (± SE) followed by the same letters within the same column are not significantly different (Tukey’s HSD test, *p* < 0.05).

## Discussion

The current study emphasizes the improved effectiveness of BEO after nanoencapsulation in a β-cyclodextrin/gum Arabic complex against *T. castaneum*. The results indicate that BEO–βCD/GA was effectively synthesized and significantly enhances the biological activity of the essential oil. In the present work, 14 compounds were identified in BEO by GC-MS analysis, with D-limonene (59.38%) being detected as the predominant compound, followed by β-myrcene (12.53%), linalool (9.4%), and trans-β-ocimene (8.61%). This result was comparable to those documented earlier^10,30^. The variations in relative concentrations may be attributed to factors such as differing soil conditions, climate, and harvesting season, which influence the phytochemical composition of the examined plants^10^.

Different approaches are available for dehydrating essential oils encapsulated into various carrier systems. Due to essential oils’ susceptibility to high temperatures, freeze-drying is deemed an appropriate technique, as it operates at low temperatures. This technology produces powders with superior rehydration characteristics and minimizes the degradation of volatile chemicals by slowing down or inhibiting degradation reactions^20^. Moreover, freeze-drying has been demonstrated to provide high product yield and effectively preserve phytochemical substances when utilizing carriers such as maltose, maltodextrin, and gum Arabic^31^.

The nanoformulations developed in this work by freeze-drying exhibited particle sizes in the nanometric range and low polydispersity indices, indicating uniformity and colloidal stability. Particle size significantly influences formulation stability and physicochemical characteristics. Additionally, the polydispersity index (PDI) is a crucial metric that reflects the uniformity of the particle size distribution in nanoencapsulated formulations^16^. In the present work, particle size was significantly affected by oil concentration, with higher concentrations (10.0% and 5.0% for F1 and F2, respectively) resulting in larger particle sizes. However, no significant difference was observed between these two formulations. On the other hand, the DLS graphs show narrow peaks, and the polydispersity index (PDI) values are very low (< 0.1) and show no significant variation across all formulations, indicating a uniform particle size distribution and good dispersion stability of the prepared inclusions^32^. In a previous study, the encapsulated lemongrass EO and its components within GA demonstrated particle sizes of 218.54 nm and 250.12 nm, respectively, and PDI values of 0.382 for the oil and 0.413 for its components^16^. Assuming that a carrier with superior emulsifying properties, such as GA, could produce such small particle sizes.

The zeta potential measurements, ranging from − 19.06 to − 31.23 mV, indicated that all synthesized BEO–βCD/GA formulations exhibited a negative surface charge. F1 showed the largest negative zeta potential (− 31.23 mV) of the studied formulations, indicating improved colloidal stability in comparison to the other formulations. Higher absolute zeta potential dispersions, whether positive or negative, generally show greater electrostatic repulsion between particles, which lessens aggregation and increases stability^33^. According to reports, sufficiently stable dispersion systems are characterized by zeta potential values near ± 30 mV^34^. As a result, the zeta potential for F1 shows a very stable formulation, whilst the values for F2 and F3 show a moderate level of stability. The current results are in line with earlier studies on encapsulation systems based on cyclodextrin. For instance, zeta potential values for β-cyclodextrin/geraniol inclusion complexes were reported to range from − 19 to − 21 mV^35^. Similarly, with cedar essential oil encapsulated in β-cyclodextrin, zeta potential values ranged from − 17.5 to − 37.0 mV, indicating the formation of stable inclusion complex dispersions^36^. Therefore, the successful production of BEO–βCD/GA inclusion complexes is confirmed by the zeta potential values reported in this investigation. While DLS, PDI, and zeta potential analyses provided evidence of particle size distribution and colloidal stability, further microscopic investigations are needed to confirm particle morphology and structural organization.

EE is a critical metric due to the necessity of preserving a high concentration of essential oil during the encapsulation process^24^. Findings of the present study indicated that the EE increased with higher oil concentrations, attaining 88.12% for F1, which contained 10.0% BEO. The encapsulation efficiency increased with increasing oil concentration, indicating a strong affinity of the β-cyclodextrin cavities and the gum Arabic matrices for hydrophobic compounds. High encapsulation efficiency is important, as it indicates the successful integration of a greater quantity of core material (BEO), hence improving both efficacy and cost-effectiveness. A prior study indicated that the achieved EE varied from 72.49% to 98.39% for saffron EO encapsulated in various combinations of β-CD and GA^17^. β-Cyclodextrin effectively encapsulates poorly water-soluble monoterpenes by displacing water molecules within its cavity, whereas GA provides less protection due to oxidative instability^37^. Combining β-CD with GA integrates β-CD’s inclusion capacity and GA’s protective film-forming properties, thereby markedly improving the encapsulation efficiency and stability of essential oils^17^. This synergistic approach offers an exceptionally efficient method for preserving and delivering monoterpenes.

The toxicity results indicated that both BEO and BEO–βCD/GA demonstrated distinct concentration- and time-dependent toxicity; however, BEO–βCD/GA consistently exhibited enhanced insecticidal efficacy, as reflected by notably elevated mortality rates and markedly reduced LC values. The wider confidence intervals for some LC_90_ estimates at shorter exposure periods are consistent with the lower mortality observed during the early stages of treatment, when the full toxic effect had not yet been reached. Employing a broader concentration range may improve the precision of LC_90_ estimation, particularly at the early exposure periods. However, the gradual decline in LC values with prolonged exposure duration further substantiates the cumulative harmful effect of the treatments. A more consistent concentration–mortality response is suggested by the steeper probit slope shown for BEO–βCD/GA after 7 days, which could be explained by the improved stability and prolonged release characteristics provided by encapsulation. Consistent with these findings, the repellent efficacy of garlic and cinnamon essential oils encapsulated in chitosan nanoparticles intensified with higher concentrations, indicating a dose-dependent response. Moreover, elevated concentrations of both pure and nanoformulated oils led to an enhancement in FDI against *T. castaneum* adults^9^. In a similar vein, it has been reported that encapsulating bergamot and geranium essential oils within PEG-based nanoparticles increases their lethal and sublethal effects against *T. castaneum* and *Rhizopertha dominica* through contact and ingestion exposure, leading to lower LC_50_ values when compared to the corresponding non-encapsulated essential oils^14^. A prior study that assessed the fumigant toxicity of *Rosmarinus officinalis* essential oil encapsulated in β-cyclodextrin and hydroxypropyl-β-cyclodextrin against *Ectomyelois ceratoniae* larvae found that encapsulation significantly improved the persistence of insecticidal activity compared to the free essential oil^38^. Increased efficacy was attributed to the cyclodextrin matrix’s protective effect, which reduced volatilization and prolonged the release of the active ingredients. The improved efficacy observed for nano-formulated essential oils may be attributed to their greater physicochemical activity compared with the pure oils. In a nano-formulated system, enhanced mobility can facilitate more efficient interaction with insect systems, leading to improved insecticidal performance. This enhanced activity is likely associated with more effective uptake either through direct contact with the insect cuticle or via ingestion, followed by subsequent penetration through the digestive tract^39^. Additionally, the enhanced efficacy of the nanoformulation can be attributed to its greater stability, reduced volatility, and controlled release of active components, which extend insect exposure^8^.

The toxicity of BEO mostly correlates with its principal terpenoid components, including limonene and linalool, which are well known for their insecticidal and repellent properties against stored product insects, acting through neurotoxic effects, disruption of neurotransmission, and interference with insect behavior^40^. These compounds may induce toxicity by altering neurotransmission via acetylcholinesterase inhibition and modulation of octopaminergic receptors, resulting in paralysis, while also possibly impairing respiration and compromising membrane integrity^41^. Results of the current study demonstrate that BEO–βCD/GA exerted a pronounced inhibitory effect on progeny production, achieving complete suppression at higher concentrations. These findings are consistent with previous studies reporting that nanoformulations of clove, anise, *Lippia sidoides*, and *Achillea santolina* essential oils exerted significant toxic effects on the development and reproductive capacity of *T.* castaneum, *R. dominica*, and *Sitophilus zeamais*^42–45^. The significant reduction in progeny emergence underscores the efficacy of nanoformulations in facilitating long-term population control, a vital component of stored-product pest management. Nanoformulations of essential oils can significantly reduce progeny production by enabling controlled, sustained release of bioactive compounds, thereby ensuring extended exposure compared with pure oils. Their small size and greater ability to spread make it easier for them to enter the insect’s cuticle and peritrophic matrix. This disrupts normal bodily functions and reproduction, making it harder for the insect to lay eggs and for larvae to survive, and lowering the number of offspring^14,46^.

The notable decrease in grain weight loss seen at higher concentrations, especially under nanoformulation treatment, indicates the potent protective impact of BEO–βCD/GA. The LC_25_, LC_50_, and LC_90_ values obtained after the 7-day treatment were used as reference concentrations to assess the long-term protective effects of the treatments during storage, and consequently, grain weight loss reflected both the cumulative impact of insect mortality and feeding activity over the 3-month period. At elevated levels, grain damage was nearly entirely neglected, indicating efficient inhibition of feeding activity. This result is further supported by the significant increase in the FDI, which was higher for the nanoformulation than for the pure oil. The enhanced antifeedant efficacy indicates that nanoencapsulation not only increases mortality rates but also amplifies behavioral disruption, including feeding inhibition and repellency. The very high feeding inhibition observed at LC_90_ of BEO–βCD/GA may be partially influenced by the increased mortality at higher concentrations, which reduces the number of actively feeding insects; however, the significant reduction in grain weight loss already observed at LC_25_ and LC_50_, where mortality remained partial, confirms that the formulation also exerts a direct antifeedant effect in addition to its toxic action. Such effects have been widely reported, where nanoformulations enhance the interaction of essential oils with insect sensory systems and negatively affect FDI compared to pure oil, for instance, against *T. castaneum*^47^, *R.dominica*^48^, and *Ephestia kuehniella*^49^.

In the present work, neither treatment negatively impacted wheat germination nor seedling vigor, even at LC_90_ concentrations. This suggests that both BEO and its nanoformulation do not compromise seed viability and can be used without harming grain quality. Maintaining germination capacity is vital for stored-grain protectants, and the current findings support the efficacy of nanoencapsulated essential oils as safe alternatives to traditional insecticides. Similar findings have been documented for nanoformulations that proficiently manage pests while preserving seed quality^2,42^. The remarkable efficacy of BEO–βCD/GA may be due to the synergistic integration of enhanced physicochemical stability, controlled release, and improved bioavailability of active compounds. These attributes result in increased toxicity, enhanced feeding deterrence, reduced offspring production, and more efficient grain protection.

## Conclusions

This study demonstrates that nanoencapsulation of bergamot essential oil (BEO) within β-cyclodextrin/gum Arabic markedly improves its physicochemical properties and biological efficacy against *T. castaneum* adults. The formulated nanoformulation exhibited high encapsulation efficiency, a uniform particle size distribution, and a relatively high negative zeta potential, all of which are indicative of a homogeneous system with favorable physicochemical stability, resulting in greater insecticidal efficacy than pure oil. BEO–βCD/GA exhibited significantly enhanced insecticidal efficacy, resulting in higher mortality rates and lower LC values across all exposure intervals. Additionally, the BEO–βCD/GA significantly inhibited progeny production, achieving maximum inhibition at higher concentrations. Moreover, it substantially reduced grain weight loss and exhibited potent antifeedant properties, indicating its dual function in inducing insect toxicity and inhibiting feeding behavior. The findings indicated that the nanoencapsulated formulation did not negatively affect wheat seed viability or germination, underscoring its suitability for practical use in stored-grain protection. However, to fully assess the agricultural safety of BEO and BEO–βCD/GA formulations, future studies should include longer evaluation periods to examine potential delayed phytotoxic effects on seed germination and early seedling development. The results indicate that β-cyclodextrin/gum Arabic-based nanoencapsulation is an efficacious method for improving the stability, delivery, and bioactivity of essential oils. This method presents a viable, environmentally sustainable alternative to traditional synthetic insecticides for the effective management of stored-product pests. Subsequent research should emphasize broader application, long-term storage stability, and field validation to enhance its practical usage. Future studies should focus on optimizing process conditions to ensure the stability and retention of volatile constituents within the encapsulated system while using economical, energy-efficient production techniques from an industrial and scale-up perspective.

## Supplementary Information

Below is the link to the electronic supplementary material.

Supplementary Material 1

## Data Availability

All data generated or analyzed during this study are included in the article.
