# Supplementary material for: Enhanced insecticidal efficacy of nanoencapsulated bergamot essential oil against stored-product pest Tribolium castaneum (Herbst)
Source: Sci Rep. 2026 Jul 17;16:22534. doi: 10.1038/s41598-026-61299-8 (PMC13379571; doi:10.1038/s41598-026-61299-8)
Supplement: Supplementary file 1 — Supplementary Material 1 [file 41598_2026_61299_MOESM1_ESM.docx]

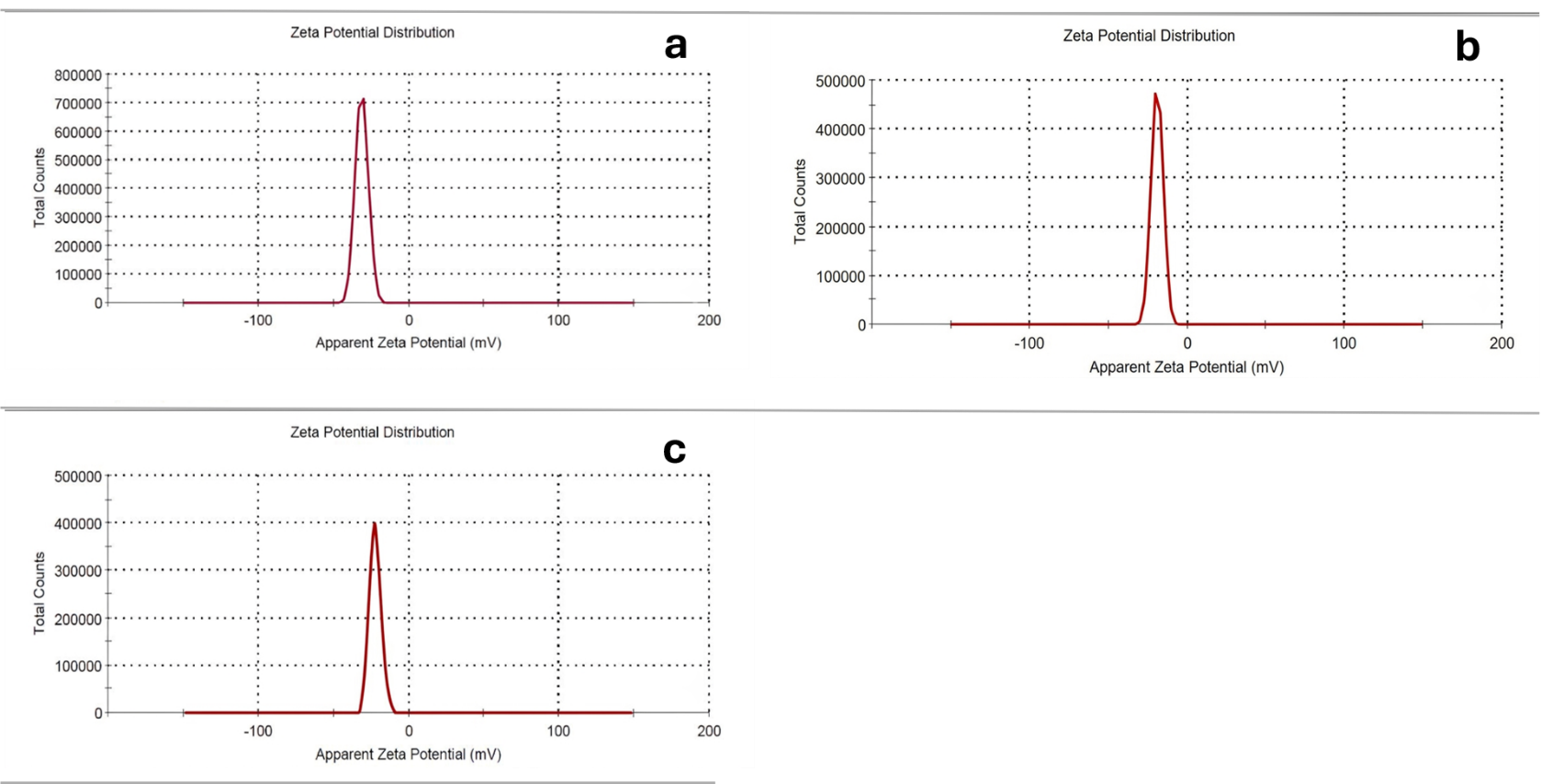


Fig. S1 Average zeta potential of different formulations (a: F1, b: F2, and c: F3) of bergamot essential oil–loaded β-Cyclodextrin/Gum Arabic (BEO–βCD/GA). F1, F2, and F3 are formulations that contain 10.0, 5.0, and 2.5% bergamot oil
